# Low prevalence of *Plasmodium falciparum* parasites lacking *pfhrp2/3* genes among asymptomatic and symptomatic school-age children in Kinshasa, Democratic Republic of Congo

**DOI:** 10.1186/s12936-022-04153-2

**Published:** 2022-04-19

**Authors:** Sabin S. Nundu, Hiroaki Arima, Shirley V. Simpson, Ben-Yeddy Abel Chitama, Yannick Bazitama Munyeku, Jean-Jacques Muyembe, Toshihiro Mita, Steve Ahuka, Richard Culleton, Taro Yamamoto

**Affiliations:** 1 Programme for Nurturing Global Leaders in Tropical and Emerging Communicable Diseases, Graduate School of Biomedical Sciences, Nagasaki University, Nagasaki, Japan; 2 Department of International Health and Medical Anthropology, Institute of Tropical Medicine, Nagasaki University, Nagasaki, Japan; 3 Institut National de Recherche Biomédicale, Kinshasa, Democratic Republic of Congo; 4 Department of Protozoology, Institute of Tropical Medicine, Nagasaki University, Nagasaki, Japan; 5 Division of Global Epidemiology, International Institute for Zoonosis Control, Hokkaido University, Sapporo, Japan; 6 Department of Tropical Medicine and Parasitology, Faculty of Medicine, Juntendo University, Tokyo, Japan; 7 Division of Molecular Parasitology, Proteo-Science Center, Ehime University, Ehime, Japan

**Keywords:** Malaria, Rapid diagnostic tests, School-age children, Democratic Republic of Congo

## Abstract

**Background:**

Loss of efficacy of diagnostic tests may lead to untreated or mistreated malaria cases, compromising case management and control. There is an increasing reliance on rapid diagnostic tests (RDTs) for malaria diagnosis, with the most widely used of these targeting the *Plasmodium falciparum* histidine-rich protein 2 (*Pf*HRP2). There are numerous reports of the deletion of this gene in *P. falciparum* parasites in some populations, rendering them undetectable by *Pf*HRP2 RDTs. The aim of this study was to identify *P. falciparum* parasites lacking the *P. falciparum* histidine rich protein 2 and 3 genes (*pfhrp*2/3) isolated from asymptomatic and symptomatic school-age children in Kinshasa, Democratic Republic of Congo.

**Methods:**

The performance of *Pf*HRP2-based RDTs in comparison to microscopy and PCR was assessed using blood samples collected and spotted on Whatman 903™ filter papers between October and November 2019 from school-age children aged 6–14 years. PCR was then used to identify parasite isolates lacking *pfhrp2/3* genes.

**Results:**

Among asymptomatic malaria carriers (N = 266), 49%, 65%, and 70% were microscopy, *Pf*HRP2_RDT, and *pfldh*-qPCR positive, respectively. The sensitivity and specificity of RDTs compared to PCR were 80% and 70% while the sensitivity and specificity of RDTs compared to microscopy were 92% and 60%, respectively. Among symptomatic malaria carriers (N = 196), 62%, 67%, and 87% were microscopy, *Pf*HRP2-based RDT, *pfldh*-qPCR and positive, respectively. The sensitivity and specificity of RDTs compared to PCR were 75% and 88%, whereas the sensitivity and specificity of RDTs compared to microscopy were 93% and 77%, respectively. Of 173 samples with sufficient DNA for PCR amplification of *pfhrp*2/3, deletions of *pfhrp2* and *pfhrp3* were identified in 2% and 1%, respectively. Three (4%) of samples harboured deletions of the *pfhrp2* gene in asymptomatic parasite carriers and one (1%) isolate lacked the *pfhrp3* gene among symptomatic parasite carriers in the RDT positive subgroup. No parasites lacking the *pfhrp2/3* genes were found in the RDT negative subgroup.

**Conclusion:**

*Plasmodium falciparum* histidine-rich protein 2/3 gene deletions are uncommon in the surveyed population, and do not result in diagnostic failure. The use of rigorous PCR methods to identify *pfhrp*2/3 gene deletions is encouraged in order to minimize the overestimation of their prevalence.

**Supplementary Information:**

The online version contains supplementary material available at 10.1186/s12936-022-04153-2.

## Background

Despite concerted control efforts, malaria remains a serious public health problem in the Democratic Republic of the Congo (DRC). The country accounted for 12% of all estimated malaria cases and 11% of deaths globally in 2019 [1]. Malaria case management is based on rapid and accurate diagnosis and prompt treatment with effective anti-malarial drugs [2].

The World Health Organization (WHO) recommends malaria diagnosis to be performed by microscopy or through the use of rapid diagnostic tests (RDTs) for all individuals presenting with malaria-like symptoms prior to the commencement of treatment [3]. However, although microscopy is the gold standard for diagnosis [4], its use is challenging and subject to both false positive and negative results when performed by inexperienced microscopists, especially in the case of poor blood film preparation and when parasitaemia is low [5–10]. RDTs are frequently used as an alternative, especially in remote areas [11–14]. In regions where *P. falciparum* is the most prevalent malaria parasite species, the most frequently used RDTs target *P*. *falciparum* histidine-rich protein-2 (*Pf*HRP2). Sixty-four percent of all RDTs distributed by national malaria control programs worldwide in 2018 were of this type [15]. Moreover, *Pf*HRP2-based RDTs have better sensitivity [16, 17] and greater thermal stability [18] than other RDTs. Furthermore, numerous antibodies used to detect *Pf*HRP2 also detect *P. falciparum* histidine-rich protein 3 (*Pf*HRP3) as they have a high degree of similarity in their amino acid sequences [19, 20]. However, the sensitivity of RDTs is dependent on the level of parasitaemia in the patient. Parasitaemia lower than 200 per μL of blood may be associated with false negative results [21]. Moreover, *pfhrp*2 and *pfhrp*3 (*pfhrp*2/3) may be deleted in some parasites rendering them undetectable by *Pf*HRRP2-based RDTs [1]. This loss of efficacy can lead to untreated or mistreated malaria cases, thus compromising malaria case management and control [17]. Thus, the WHO recommends continuous nationwide surveillance of parasites harbouring *pfhrp*2/3 deletions. It is recommended that if their prevalence exceeds 5%, alternative RDTs should be used [1]. In the DRC, the 2013–2014 nationwide demographic and health survey revealed a *pfhrp*2 gene deletion prevalence of 6.4% overall and 21.9% in Kinshasa among asymptomatic under five children [22]. Interestingly, no *pfhrp*2/3 gene deletions were detected among symptomatic individuals [23]. Munyeku et al. [24], found an overall prevalence of 9.2% of parasites isolated from symptomatic malaria patients living Kwilu province, (near Kinshasa) carried *Pfhrp2* gene deletions. However, only 9.9% of isolates that gave false negative *Pf*HRP2-based RDTs results in that study carried *pfhrp*2 gene deletions, suggesting that the vast majority of RDT failures are not due to *pfhrp2* gene deletions in that region. A previous survey conducted in 2011, that included 133 asymptomatic children in the Mont-Ngafula-2 health zone (HZ) and 145 asymptomatic children in the Selembao HZ aged 6–59 months found a prevalence of 35% and 27%, respectively, when tested by RDT [25]. A study conducted in the same two areas in 2019 and including 427 asymptomatic and 207 symptomatic school-aged children aged 6–14 years found 41% (Mont-Ngafula-2: 56%; Selembao: 28%) and 64% (Mont-Ngafula-2: 66%; Selembao: 63%) of malaria prevalence by RDT, respectively [26].

This study aimed to assess the prevalence of *P. falciparum* parasites lacking the *pfhrp*2/3 genes in isolates from asymptomatic and symptomatic school-age children in Kinshasa.

## Methods

### Study design, study area and selection of participants

**Fig. 1:**
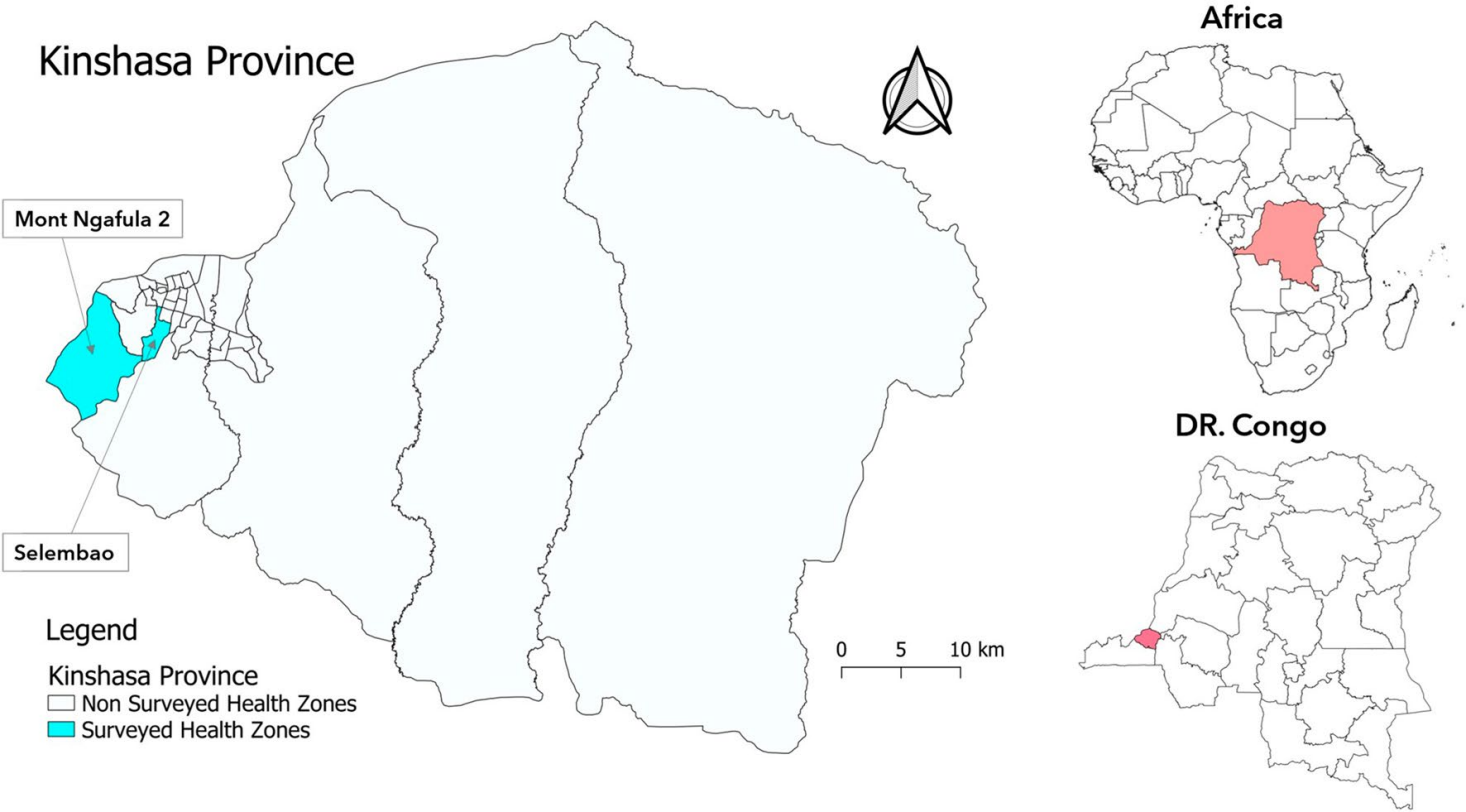
Sample collection sites

Samples used in this study were collected from a previous cross-sectional survey carried out in October and November 2019 among school-age children with ages ranging between 6 and 14 years in Mont-Ngafula-2 rural health zone (HZ) and Selembao urban HZ of Kinshasa, Democratic Republic of Congo (Fig. 1) [26].

634 school-age children were enrolled in the study (427 asymptomatic and 207 symptomatic). Finger-prick blood were collected from each child between October and November 2019 for *Pf*HRP2-based RDT diagnosis (5 µL of blood), microscopy, and for the preparation of blood spots on Whatman 903™ filter paper (three drops of capillary blood). DNA were extracted and kept at − 80 °C until use. Nested-PCR targeting the *Plasmodium* mitochondrial cytochrome c oxidase III (Cox3) gene was performed for identification of *Plasmodium* species (266 asymptomatic and 196 symptomatic samples were analysed) as described in a previous report [26]. Asymptomatic schoolchildren not showing fever and/or malaria-related symptoms, including headache, chills, body joint pains, fatigue, 2 weeks prior to the survey were recruited from schools. Symptomatic children were recruited from health facilities and were outpatients seeking healthcare due to fever or/and malaria-related symptoms within 72 h prior to the survey. Schoolchildren whose parents or relatives signed written consent forms were included in this study [26]. Four hundred and sixty-two positive DNA samples (210 microscopy negative, 252 microscopy positive and 157 *Pf*HRP2 RDT negative, 305 *Pf*HRP2 RDT positive) were used in this study for assessment of *pfhrp*2/3 gene deletions.

### Detection of *P. falciparum* infection and selection of samples for *pfhrp2/3* PCR

Real-time PCR (qPCR) targeting the *P. falciparum* lactate dehydrogenase gene (*pfldh*) was performed to quantify the number of parasite genomes per µL of extracted DNA solution from each of the samples using a serial dilution of laboratory cultured *P. falciparum* 3D7 strain DNA for calibration. Excluding samples with DNA concentrations less than the limit of detection (LOD) of the *pfhrp2/3* PCR is crucial for the avoidance of false negative results. A serial dilution consisting of 0.1, 0.01, 0.001 and 0.0001 ng/µL of gDNA extracted from cultured *P. falciparum* 3D7 was prepared in order to generate a calibration curve [23, 27].

### *pfldh* qPCR for selection of samples with sufficient DNA for pfhrp2/3 PCR

**Fig. 2:**
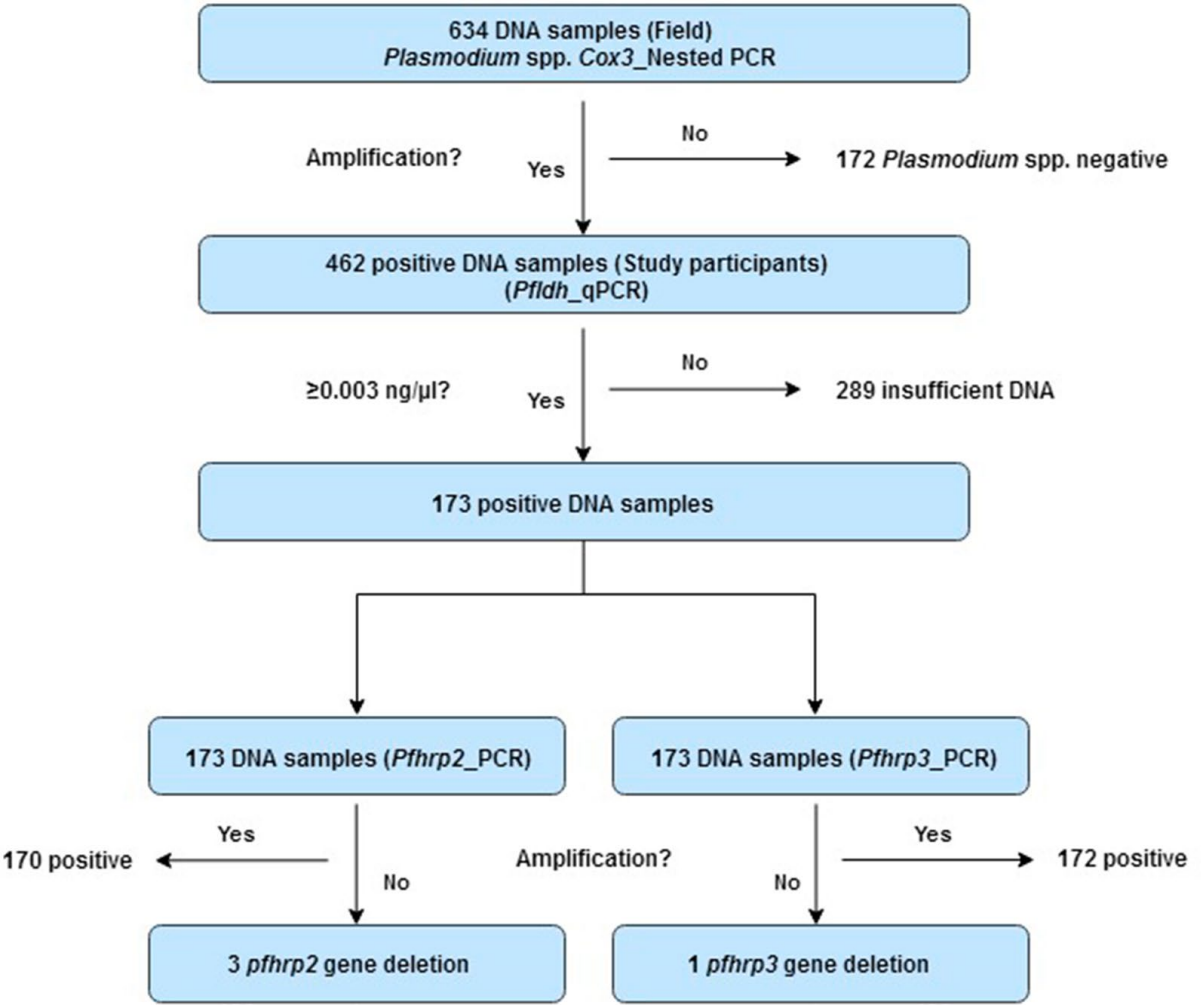
Assessment of *pfhrp*2/3 gene deletion

The LOD of the *pfhrp2* and *pfhrp3* PCR assays used in this study was 1 × 10^–3^ ng/µL. In order to ensure that only samples with sufficient DNA for the amplification of *pfhrp2* and *pfhrp3* were used, only samples with greater than 3 × 10^–3^ ng/µL of DNA as determined by *pfldh* qPCR were considered for further analysis (Additional file 1: Table S1) [23, 27] (Fig. 2).

A calibration curve was prepared using the results of qPCR with control samples (0.1 ng/μL, 0.01 ng/μL, 0.001 ng/μL and 0.0001 ng/μL). Duplicated samples were loaded in 96-wells plates along with serially diluted positive controls (using gDNA extracted from cultured *P. falciparum* 3D7) as well as negative controls consisting of DNA samples from known malaria negative individuals (RDT-, microscopy- and PCR-) and distilled water for checking contamination. The assay was repeated for all discordant duplicates and three consistent results were required for confirmation. The DNA concentration of samples were quantified from each Ct values and the calibration curve.

For selection of samples for *pfhrp2/3* PCR, all samples were duplicated, and loaded in 96-wells plates along with positive and negative controls as described above using LightCycler® 480 SYBR Green I Master, 200 nM of forward primer (5′-ACGATTTGGCTGGAGCAGAT-3′), 200 nM of reverse primer (5′-TCTCTATTCCATTCTTTGTCACTCTTC-3′) and Template DNA (1 µL) with 12 µL of total volume. The thermal cycling conditions were 50 °C for 2 min, 95 °C for 10 min, and 50 cycles of 95 °C for 15 s and 60 °C for 1 min, 95 °C for 5 s, 65 °C for 1 min, and 97 °C for 5 s (Additional file 1: Table S1) [27]. The threshold cycle (CT) value set was the same for all reactions. The LOD of the *pfldh* qPCR assays used for selection of samples for *pfhrp2/3* PCR was ≥ 3 × 10^–3^ ng/µL of DNA.

### Detection of *pfhrp2/3* gene deletions

*Pfhrp2* and *pfhrp3* PCR genotyping was performed as previously described [26], with minor modifications using conventional single step PCR with primers targeting exon 2 of the genes. Selected samples were used to amplified *pfhrp2* PCR using One *Taq* 2× Master Mix with standard buffer, DNA template (3 μL), 400 nM of forward primer (5′-CAAAAGGACTTAATTTAAATAAGAG-3′), 400 nM reverse primer (5′-AATAAATTTAATGGCGTAGGCA-3′) in a 25 µL final volume. *pfhrp3* PCR was performed using One *Taq* 2× Master Mix with standard buffer, DNA template (3 μL), 400 nM of forward primer (5′-AATGCAAAAGGACTTAATTC-3′), 400 of nM reverse primer (5′-TGGTGTAAGTGATGCGTAGT-3′) in a 25 µL final volume with reaction conditions 95 °C for 10 min and 45 cycles of 94 °C for. 50 s, 55 °C for 50 s and 70 °C for 1 min (Additional file 1: Table S1) [27]. Genomic DNA from 3D7 (*pfhrp2/3* positive), Dd2 (*pfhrp2* negative) and HB3 (*pfhrp3* negative) were used as controls. PCR products were visualized under UV light on 1.5% agarose gels run at 100 V for 30 min and stained with Gel Red® solution (Biotium. California, USA) for 30 min.

### Statistical analyses

Data was analysed using STATA version 14.2 (College Station. Texas, USA). Descriptive variables are presented as proportions (categorical variables) or median and interquartile range (continuous variables). Chi-square tests (or Fisher’s exact tests when appropriate) were used to assess associations between categorical variables and *pfhrp2/3* gene deletion prevalence. Sensitivity (= true positive/(true positive + false negative), specificity (= true negative/(true negative + false positive), Positive predictive value (= true positive/(true positive + false positive) and negative predictive value (= true negative/(true negative + false negative) of RDTs were calculated using PCR and microscopy as the gold standard. Agreement between diagnostic techniques was assessed using Cohen’s kappa coefficient. The sensitivity and the specificity of RDTs and microscopy at densities between 1 × 10^–4^ ng/μL and 3 × 10^–3^ ng/μL and those greater than 3 × 10^–3^ ng/μL of extracted DNA was assessed [27]. P-values of below 0.05 were considered significant.

## Results

### Socio-demographic characteristics of the participants and malaria diagnosis

462 school-age children, of which 266 were asymptomatic, and 196 were symptomatic were enrolled. Of the 266 asymptomatic children, 136/266 (51%) were female, 147/266 (55%) were between the ages of 6 and 9 and 168/266 (63%) lived in rural areas. Of the 196 symptomatic children, 94/196 (48%) were female, 132/196 (67%) were between the ages of 6 and 9 and 102/196 (52%) lived in rural areas (Table 1).

**Table 1:** Socio-demographic characteristics of asymptomatic (N = 266) and symptomatic (N = 196) participants

Variables	Number (%)
Asymptomatic infection	
Sex	
Female	136 (51)
Male	130 (49)
Age med. (IQR)	9 (7–11)
6–9	147 (55)
10–14	119 (48)
Location	
Rural	168 (63)
Urban	98 (37)
Symptomatic infection	
Sex	
Female	94 (48)
Male	102 (52)
Age med. (IQR)	8 (7–11)
6–9	132 (67)
10–14	64 (33)
Location	
Rural	102 (52)
Urban	94 (48)

*IQR* interquartile range, *med.* median

### Comparison of RDT with PCR and microscopy

Among 266 DNA samples from asymptomatic children, 174/266 (65%), 187/266 (70%) and 130/266 (49%) were *Pf*HRP2_RDT, *pfldh*-qPCR and microscopy positive, respectively. The sensitivity and specificity of RDTs compared to PCR were 150/187 (80%; 95% CI 74, 86) and 55/79 (70%, 95% CI 58, 80) while the sensitivity and specificity of RDTs compared to microscopy were 119/130 (92%, 95% CI 85, 96) and 81/136 (60%, 95% CI 51, 68), respectively. Agreement between *Pf*HRP2-based RDTs and PCR was moderate (Cohen’s kappa = 0.48) as was the agreement between *pfhrp*2-based RDTs and microscopy (Cohen’s kappa = 0.51) (Table 2).

**Table 2:** *Pf*HRP2_RDT performance compared to PCR and microscopy examination in asymptomatic (N = 266) and symptomatic (N = 196) infections

*Pf*HRP2_RDTs	Asymptomatic infections					
	*Pfldh*_qPCR			Microscopy		
	Positive	Negative	Total	Positive	Negative	Total
Positive	150	24	174	119	55	174
Negative	37	55	92	11	81	92
Total	187	79	266	130	136	266
Se (%) (CI 95%)	80 (74, 86)			92 (85, 96)		
Sp (%) (CI 95%)	70 (58, 80)			60 (51, 68)		
PPV (%) (CI 95%)	86 (81, 90)			84 (81, 87)		
NPV (%) (CI 95%)	60 (52, 68)			75 (63, 84)		
Kappa*	0.48, p < 0.001			0.51, p < 0.001		

*Pf*HRP2_RDTs	Symptomatic infections					
	*Pfldh*_qPCR			Microscopy		
	Positive	Negative	Total	Positive	Negative	Total
Positive	128	3	131	114	17	131
Negative	43	22	65	8	57	65
Total	171	25	196	122	74	196
Se (%) (CI 95%)	75 (68, 81)			93 (88, 97)		
Sp (%) (CI 95%)	88 (69, 98)			77 (66, 86)		
PPV (%) (CI 95%)	98 (94, 99)			97 (95, 98)		
NPV (%) (CI 95%)	34 (28, 41)			64 (47, 78)		
Kappa*	0.37, p < 0.001			0.72, p < 0.001		

*Se* sensitivity, *Sp* specificity, *PPV* positive predictive value, *NPV* negative predictive value, *CI* confidence interval

*Statistical analysis using Cohen’s kappa coefficient test, significance at p < 0.05

Among 196 DNA samples from symptomatic infections, 131/196 (67%), 171/196 (87%) and 122/196 (62%) were *Pf*HRP2-based RDTs, *pfldh*-qPCR and microscopy positive, respectively. The sensitivity and specificity of RDTs compared to PCR were 128/171 (75%, 95% CI 68, 81) and 22/25 (88%, 95% CI: 69, 98) while sensitivity and specificity of RDTs compared to microscopy were 114/122 (93%, 95% CI 88, 97) and 57/74 (77%, 95% CI 66, 86), respectively. Findings showed satisfactory agreement between *Pf*HRP2-based RDTs and microscopy (Cohen’s kappa = 0.72) and fair agreement between *Pf*HRP2-based RDTs and PCR (Cohen’s kappa = 0.37) (Table 2).

### Performance of RDT and microscopy examinations based on parasite densities

The sensitivity of RDTs and microscopy at lower limits of parasite density below 3 × 10^–3^ ng/µL of extracted DNA, and those above 3 × 10^–3^ ng/µL were compared. The sensitivity and specificity of RDTs were 96% (95% CI 92, 98) (symptomatic: 93% (87, 97); asymptomatic: 100% (95, 100) and 37% (95% CI 31, 45) [symptomatic: 55% (42, 67); asymptomatic: 31% (23, 40)] while the sensitivity and specificity of microscopy were 91% (symptomatic: 90%; asymptomatic: 94%) and 59% (symptomatic: 65%; asymptomatic: 56%) (Table 3).

**Table 3:** Sensitivity and specificity of RDTs and microscopy based on *P. falciparum* DNA concentrations measured by qPCR (N = 358), in asymptomatic (N = 187) and symptomatic (N = 171) infections

DNA concentration	Overall					
	RDTs			Microscopy		
	Positive	Negative	Total	Positive	Negative	Total
1 × 10^–4^–3 × 10^–3^ ng/µL	112	73	185	75	110	185
≥ 3 × 10^–3^ ng/µL	166	7	173	158	15	173
Total	278	80	358	233	125	358
Se (%) (CI 95%)	96 (92, 98)			91 (86, 95)		
Sp (%) (CI 95%)	37 (31, 45)			59 (52, 67)		
PPV (%) (CI 95%)	84 (83, 86)			89 (87, 90)		
NPV (%) (CI 95%)	73 (56, 85)			67 (55, 77)		

DNA concentration	Asymptomatic infections					
	RDTs			Microscopy		
	Positive	Negative	Total	Positive	Negative	Total
1 × 10^–4^–3 × 10^–3^ ng/µL	82	37	119	52	67	119
≥ 3 × 10^–3^ ng/µL	68	0	68	64	4	68
Total	150	37	187	116	71	187
Se (%) (CI 95%)	100 (95, 100)			94 (86, 98)		
Sp (%) (CI 95%)	31 (23, 40)			56 (47, 65)		
PPV (%) (CI 95%)	78 (75, 80)			84 (81, 86)		
NPV (%) (CI 95%)	100			80 (61, 91)		

DNA concentration	Symptomatic infections					
	RDTs			Microscopy		
	Positive	Negative	Total	Positive	Negative	Total
1 × 10^–4^–3 × 10^–3^ ng/µL	30	36	66	23	43	66
≥ 3 × 10^–3^ ng/µL	98	7	105	94	11	105
Total	128	43	171	117	54	171
Se (%) (CI 95%)	93 (87, 97)			90 (82, 95)		
Sp (%) (CI 95%)	55 (42, 67)			65 (54, 77)		
PPV (%) (CI 95%)	93 (91, 95)			95 (93, 96)		
NPV (%) (CI 95%)	55 (36, 72)			48 (34, 62)		

*Se* sensitivity, *Sp* specificity, *PPV* positive predictive value, *NPV* negative predictive value, *CI* confidence interval

### Detection of *pfhrp2/3* gene deletions

A conservative criterion for the detection of *pfhrp*2/3 gene deletions was used through the selection of samples with DNA concentrations three times higher than the limit of detection of the *pfhrp2/3* PCR assays. Of 462 DNA samples, 173 were selected for *pfhrp2/3* PCR analysis following *pfldh* qPCR. Of the 173 isolates used for *pfhrp*2/3 PCR, three were *pfhrp*2 negative and one was *pfhrp*3 negative (Fig. 2).

The overall prevalence of the *pfhrp2* gene deletion was 2% (3/173) while it was 1% (1/173) for the *pfhrp3* gene. All four samples that contained these mutant parasites had returned positive RDT results. Only 7 RDT negative samples had sufficient parasite densities for *pfhrp2/3* deletion, and none of these had *pfhrp*2/3 gene deletions (Table 4).

**Table 4:** Prevalence of *pfhrp*2/3 gene deletion based on *Pf*HRP2_RDT results (N = 173)

RDTs	Pfhrp2_PCR			Pfhrp3_PCR		
	Positive	Negative	Total	Positive	Negative	Total
	n (%)	n (%)	n (%)	n (%)	n (%)	n (%)
Positive	163 (98)	3 (2)	166 (100)	165 (99)	1 (1)	166 (100)
Negative	7 (100)	0 (0)	7 (100)	7 (100)	0 (0)	7 (100)
Total	170 (98)	3 (2)	173 (100)	172 (99)	1 (1)	173 (100)

### Prevalence of *phrp2/3* gene deletion by age, sex, health status and location

Among the three samples that harboured *pfhrp*2 gene deletions, two were from children aged 6 to 9 years, and all three were from female children, asymptomatic individuals and children living in the urban area. Age, sex, children health status and location were not associated to *phhrp2/3* gene deletion. No significant associations were found between *pfhrp*2/3 prevalence and age, sex, health status and location (p > 0.05, Additional file 1: Table S2).

## Discussion

Malaria rapid diagnostic tests play an important role in malaria case management and surveillance. Based on several reports that assessed the prevalence of *pfhrp*2/3 gene deletions, the WHO has recently recommended continuous surveillance of *Pfhrp2/3-*deleted *P. falciparum* [17, 28, 29]. This study used a rigorous method of DNA sample selection for evaluation of *Pfhrp2/3-*deleted *P. falciparum* [23, 27], which minimizes the overestimation of *pfhrp2/3-*deleted *P. falciparum* that may occur through conventional approaches [22, 30, 31]. It is important to consider DNA quantity in samples subjected to PCR to identify *pfhrp2/3* deletions, as low DNA levels may lead to false *pfhrp2*-negative results and overestimation of the prevalence of *pfhrp2/3* gene deletions.

Three isolates harbouring a *pfhrp2* gene deletion and one isolate harbouring a *pfhrp*3 gene deletion were found among *pfhrp*2-based RDT positive samples. The two *pfhrp2* negative samples were presumably positive by *pfhrp*2-based RDT due to cross reaction with PfHRP3 [20, 32, 33]. The sample harbouring a *pfhrp*3 gene deletion was from a symptomatic child while the three samples harbouring *pfhrp*2 gene deletions were from asymptomatic children. It has been shown that *pfhrp*2/3*-*deleted parasites do not differ from wild-type parasites in their ability to cause malaria symptoms [34]. Previous studies conducted in the DRC have found a *pfhrp2* gene deletion prevalence of 6.4% across the country and 21.9% in Kinshasa in a nationwide demographic and health survey among asymptomatic children [22] and 9.2% amongst symptomatic individuals in a neighbouring province of Kinshasa [24]). This difference may be explained by different methods used for the detection of *Pfhrp2/3* deletions. A previous study conducted in the DRC using a similar method of selection of samples with sufficient parasite DNA for the detection of *Pfhrp*2/3 gene deletions, did not find any isolates harbouring *pfhrp2/3-*deletions among symptomatic children [23] highlighting the fact that the method used in the previous large survey of asymptomatic parasite carriers [22] may have overestimated the prevalence of the *pfhrp2* gene deletion.

Seven isolates were negative by RDT, but positive by qPCR with over 3 × 10^–3^ ng of parasite DNA per µL of extracted DNA solution. Five of these samples were negative by microscopy, suggesting relatively low parasitaemia. RDT failure in these cases may be explained by data recording errors, operator-dependent and manufacturing quality [35–37] or by the presence of anti-*pfhrp*2 antibodies binding to the circulating antigens [38] or possibly due to the presence of mixed infection *pfhrp*2-negative and *pfhrp*2-positive parasites in the same isolates [39].

Among 196 isolates from symptomatic children, the sensitivity of *Pf*HRP2-based RDTs compared to *pfldh*-qPCR was 75%. Of 43 *pfhrp2* RDT negative PCR positive isolates, 36 (84%) had lower than 3 × 10^–3^ ng/µL of extracted DNA, highlighting the fact that RDTs are less sensitive at low parasitaemia compared to PCR [21]. This may exclude some symptomatic children from treatment [26].

Among 266 isolates from asymptomatic children, the sensitivity of *Pf*HRP2-based RDTs compared to *pfldh*-qPCR was 82%. All 37 RDT negative PCR positive isolates had below 3 × 10^–3^ ng/µL solution, highlighting the importance of the use of PCR for the diagnosis of asymptomatic malaria parasite carriers [26, 40–44]. However, for malaria case management, PCR may be prohibitively expensive, time-consuming and technically challenging especially in remote locations [45, 46]. There is a need to develop a more cost-effective highly sensitive malaria diagnostic test suitable for remote areas [45].

Although the samples used in this study may not be representative of the country as a whole, the method used minimized overestimation of the prevalence of *P. falciparum* parasites carrying *pfhrp2/3-*deletions, which may occur with conventional methods.

## Conclusion

The prevalence of *P. falciparum* parasites carrying deletions of the *pfhrp2/3* gene is low in the population surveyed in this study, suggesting the use of *Pf*HRP2-based RDTs remains appropriate for the detection of malaria in this region. The continuous use of rigorous PCR methods for surveys of *pfhrp*2/3 gene deletion prevalence is, therefore, encouraged.

## Supplementary Information

**Additional file 1: Table S1.** Primer sequences and PCR conditions for *P. falciparum ldh*, *hrp*2/3 PCR amplification. **Table S2.** Prevalence of *P. falciparum hrp2/3* gene deletion by age, sex, health status and location (N = 173).

## Data Availability

The datasets used and/or analysed during the current study are available from the first author (SSN).
