# Supplementary material for: Low prevalence of Plasmodium falciparum parasites lacking pfhrp2/3 genes among asymptomatic and symptomatic school-age children in Kinshasa, Democratic Republic of Congo
Source: Malar J. 2022 Apr 19;21:126. doi: 10.1186/s12936-022-04153-2 (PMC9020024; doi:10.1186/s12936-022-04153-2)
Supplement: Supplementary file 1 — Additional file 1: Table S1. Primer sequences and PCR conditions for P. falciparum ldh, hrp2/3 PCR amplification. Table S2. Prevalence of P. falciparum hrp2/3 gene deletion by age, sex, health status and location (N = 173). [file 12936_2022_4153_MOESM1_ESM.docx]

ADDITIONAL FILE 1

**Table S1. Primer sequences and PCR conditions for *P. falciparum* *ldh*, *hrp*2/3 PCR amplification**

|  |  |  |  |  |
| --- | --- | --- | --- | --- |
|  |  |  |  | **LOD, ng/μL** |
| **Target gene** | **Primer sequence (5' to 3')** | **Reaction components** | **Cycling conditions** |  |
|  |  |  | 50℃: 2min |  |
| ***Pfldh*** |  | LightCycler® 480 SYBR Green I Master : 6μL | 95℃: 10min |  |
| (initial qPCR) | For: ACGATTTGGCTGGAGCAGAT | Forward primer (2.4μM): 1μL | 95℃: 15s | 10^-4^ |
|  | Rev TCTCTATTCCATTCTTTGTCACTCTTC | Reverse primer (2.4μM): 1μL | 60℃: 1min x 50 cycles |  |
|  |  | RNase free water: 3μL | 95℃: 5s |  |
|  |  | Template DNA: 1μL | 65℃: 1min |  |
|  |  | 15uL reaction volume (Primer concentration : 200 nM) | 97℃: 5s |  |
|  |  |  |  |  |
|  | For: CAAAAGGACTTAATTTAAATAAGAG | One *Taq* 2X Master Mix with standard buffer: 12.5μL |  |  |
| ***Pfhrp2*** | Rev: AATAAATTTAATGGCGTAGGCA | 10μM forward primer: 1μL | 94˚C/10min; |  |
|  |  | 10μM reverse primer: 1μL | 45 cycles of 94˚C/50s, |  |
|  |  |  | 55˚C/50s and 70˚C/1min; |  |
|  |  | Nuclease free water: 7.5μL | 4˚ C -∞ | 10^-3^ |
| ***Pfhrp3*** | For: AATGCAAAAGGACTTAATTC | DNA template: 3μL |  |  |
|  | Rev: TGGTGTAAGTGATGCGTAGT | 25uL reaction volume (Primer concentration: 400 nM) |  |  |

**LOD; lower limit of detection**

**Table S2. Prevalence of *P. falciparum* *hrp2/3* gene deletion by age, sex, health status and location (N=173)**

|  |  |  |  |  |  |  |  |
| --- | --- | --- | --- | --- | --- | --- | --- |
|  |  | ***Pfhrp2*** |  |  | ***Pfhrp3*** |  |  |
| **Variables** |  | Negative | Positive |  | Negative | Positive |  |
|  | n | no. (%) | no. (%) | p-value | no. (%) | no. (%) | p-value |
| **Age (years)** |  |  |  | 1 |  |  | 1 |
| 6-9 | 121 | 2 (1) | 119 (98) |  | 1 (1) | 120 (99) |  |
| 10-14 | 52 | 1 (1) | 51 (98) |  | 0 (0) | 52 (100) |  |
| **Sex** |  |  |  | 0.09 |  |  | 0.45 |
| Female | 78 | 3 (4) | 75 (96) |  | 1 (1) | 77 (99) |  |
| Male | 95 | 0 (0) | 95 (100) |  | 0 (0) | 95 (100) |  |
| **Health status** |  |  |  | 0.06 |  |  | 1 |
| Asymptomatic | 68 | 3 (4) | 65 (96) |  | 0 (0) | 68 (100) |  |
| Symptomatic | 105 | 0 (0) | 105 (100) |  | 1 (1) | 104 (99) |  |
| **Location** |  |  |  | 0.09 |  |  | 0.45 |
| Rural | 96 | 0 (0) | 96 (100) |  | 0 (0) | 96 (100) |  |
| Urban | 77 | 3 (4) | 74 (96) |  | 1 (1) | 76 (99) |  |
